# Baseline Functional Connectivity of the Mesolimbic, Salience, and Sensorimotor Systems Predicts Responses to Psychological Therapies for Chronic Low Back Pain With Comorbid Depression: A Functional MRI Study

**DOI:** 10.1002/brb3.70380

**Published:** 2025-02-28

**Authors:** Sonia Medina, Carlos G. Forero, Juan P. Sanabria‐Mazo, Carla Rodríguez‐Freire, Jaime Navarrete, Owen G. O'Daly, Matthew A. Howard, Juan V. Luciano

**Affiliations:** ^1^ Department of Neuroimaging King's College London London UK; ^2^ Exeter Medical School University of Exeter Exeter UK; ^3^ School of Medicine Universitat Internacional de Catalunya Sant Cugat del Vallès Spain; ^4^ Teaching, Research & Innovation Unit Parc Sanitari Sant Joan de Déu Sant Boi de Llobregat Spain; ^5^ CIBER of Epidemiology and Public Health (CIBERESP) Madrid Spain; ^6^ Department of Clinical & Health Psychology Autonomous University of Barcelona Bellaterra Spain

**Keywords:** ACT, BATD, chronic pain, depression, fMRI

## Abstract

**Introduction:**

Chronic low back pain (CLBP) is a prevalent and debilitating condition. Cognitive behavioral therapy (CBT) can improve coping mechanisms for CLBP and pain‐related outcomes. However, the mechanisms by which they do so remain undetermined. We explored the neural correlates of CLBP symptoms and CBT action using functional magnetic resonance imaging (fMRI) in women with CLBP and comorbid depression.

**Methods:**

Forty individuals underwent fMRI followed by 8 weeks of either treatment as usual (TAU) or one of two CBT in addition to TAU: acceptance and commitment therapy (ACT) or behavioral activation treatment for depression (BATD). Pain intensity, depression, psychological inflexibility, and pain catastrophizing scores were obtained at baseline and follow‐up. Functional connectivity (FC) patterns of the salience network (SN), sensorimotor network (SMN), and the mesolimbic pathway (MLP), derived from resting‐state fMRI examination were correlated with both baseline and delta (baseline—follow‐up) pain‐related psychological measures.

**Results:**

Individuals receiving ACT and BATD showed reduced depression, psychological inflexibility, and pain catastrophizing. Strong baseline connectivity of the SN and SMN corresponded with higher pain intensity, but strong connectivity of the MLP and precuneus corresponded with lower pain intensity. Pain intensity changes correlated with mesolimbic‐salience connectivity following ACT, and with sensorimotor connectivity following BATD. Specifically, stronger baseline FC between the MLP and posterior insula predicted greater pain intensity reduction with ACT, while stronger FC between the SMN and secondary somatosensory cortex predicted greater pain intensity reduction with BATD. FC of the SN correlated with changes in psychological inflexibility across both therapies.

**Conclusions:**

We illustrate the potential of FC as a biomarker of CLBP plus depression and the response to CBT. Our data suggest ACT and BATD have differing underlying brain mechanisms. These findings indicate that FC biomarkers could guide personalized treatment, improving individual outcomes.

## Graphical abstract

Resting‐state fMRI revealed that baseline functional connectivity (FC) in the salience network and sensorimotor network (SMN) correlated with higher pain, while mesolimbic pathway connectivity correlated with lower pain. Post‐treatment, acceptance and commitment therapy (ACT) was associated with changes in mesolimbic‐salience FC, and behavioral activation treatment for depression (BATD) with changes in sensorimotor FC.

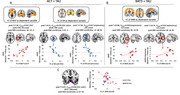

## 1 Introduction

Chronic low back pain (CLBP) affects approximately 11% of individuals, significantly impairing daily functioning (Balagué et al. 2012). The limited efficacy of pharmacological treatments stems from lacking knowledge of its pathogenesis and pathophysiology (Park et al. 2018). Comorbid depression complicates management (Gore et al. 2012), worsening prognosis (Nordstoga et al. 2017) and increasing the risk of transitioning from acute to chronic pain (Pincus et al. 2002). Therefore, psychological therapies are often included to address comorbidities and improve pain management (Last and Hulbert 2009).

New forms of cognitive behavioral therapy (CBT), such as acceptance and commitment therapy (ACT), focus on accepting pain and addressing maladaptive behaviors such as inactivity and catastrophizing. Another example is behavioral activation treatment for depression (BATD), which emphasizes engaging in valued behaviors to elicit positive reinforcement instead of avoiding anticipated suffering (Ekers et al. 2014). Furthermore, growing evidence suggests these therapies can be optimized to manage pain‐related symptoms (Saragiotto et al. 2017; Barrett et al. 2021; Mazzucchelli and Da Silva 2016). Understanding their mechanisms, particularly in responders, could explain chronic pain pathophysiology. For instance, examining brain connectivity changes linked to clinical outcomes could identify brain features as potential biomarkers for individualized treatment (Gilpin et al. 2017).

Neuroimaging has advanced our understanding of chronic pain (Martucci et al. 2014), depression (Buch and Liston 2021), and CBT mechanisms (Porto et al. 2009). Key regions associated with chronic pain (Ferraro et al. 2022) and depression (Dunlop and Mayberg 2022) include the anterior insula (AI) and the anterior cingulate cortex (ACC), forming the salience network (SN), which is a homeostatic system that directs attention to relevant stimuli with emotional valence (Seeley 2019). The SN is connected to subcortical areas involved in emotional and autonomic processing within the mesolimbic pathway (MLP), including the striatum, thalamus, and brainstem nuclei (McCutcheon et al. 2019), as well as with cortical regions responsible for somatosensory and motor processing, forming the sensorimotor network (SMN) (Kolesar et al. 2017). Moreover, a recent review suggests that the SN and the SMN mediate catastrophizing thoughts, which enhances the perceived pain of stimuli (Galambos et al. 2019). Taken together, dysregulation of communication between these networks may underlie CLBP (Lu et al. 2016) and depression (Peters et al. 2016). Such dysregulation might be partially reversible via CBTs, which aim to improve psychological inflexibility, possibly mediated by the AI, ACC, and dorsolateral prefrontal cortex (dlPFC) (Dajani and Uddin 2015). Existent literature has suggested that CBT may alter brain functional connectivity (FC) in depression (McGrath et al. 2013), phobia, obsessive‐compulsive disorder (Linden 2006), and pain conditions (Bao et al. 2022), although findings remain inconsistent. Thus, a more comprehensive theory of how CBT modulates FC in CLBP is still needed.

This study had two aims: First, to investigate brain mechanisms underlying key symptoms of CLBP plus depression (namely subjective pain intensity, depression, pain catastrophizing, and psychological inflexibility to pain), focusing on the SN, SMN, and MLP; and second, to explore relationships between baseline FC and treatment outcomes, to assess the predictive potential of rs‐fMRI's for ACT and BATD responses, and to provide insights into treatment mechanisms. We hypothesized that: (i) CLBP individuals would show stronger SN‐SMN FC and weaker SN‐MLP FC compared to controls; (ii) the stronger the connectivity between the SN and SMN, the less individuals would benefit from ACT and BATD, as catastrophizing thoughts may hinder avoidance reappraisal; (iii) SMN FC would correlate with BATD response and pain catastrophizing; and (iv) SN FC would be related to changes in psychological inflexibility.

## 2 Materials and Methods

### 2.1 Design

This study was part of a 12‐month multicenter, single‐blind randomized controlled trial (RCT) with three arms: treatment as usual (TAU) only, ACT + TAU, and BATD + TAU. This RCT was registered on ClinicalTrials.gov (NCT04140838) and followed the guidelines issued by the “Standard Protocol Items: Recommendations for Interventional Trials” (SPIRIT) and the “Consolidated Standards of Reporting Trials” (CONSORT) (Schulz et al. 2010). Initially designed to deliver the therapies in a face‐to‐face format, it was adapted to be delivered via a remote, synchronous, videoconferencing platform. The study was conducted following the Declaration of Helsinki of 1964 and was approved by the Ethics Committee of the Fundació Sant Joan de Déu (PIC‐178‐19) and the Hospital del Mar (2019/8866/I). Participants did not receive financial incentives. A detailed description of the study protocol can be found elsewhere. Data supporting the findings are available on request from the corresponding author.

### 2.2 Participants

A total of 40 women were included in this neuroimaging sub study from 234 participants in the IMPACT study at the Parc Sanitari Sant Joan de Déu (St. Boi de Llobregat, Spain) and Hospital del Mar (Barcelona, Spain). Inclusion criteria were: (i) age 18–70 years, diagnosed with CLBP for at least 3 months; (ii) pain intensity > 4/10 on a numeric rating scale (NRS) in the past week; (iii) moderate to severe depressive symptoms according to Patient Health Questionnaire (PHQ‐9) (McMillan et al. 2010) (total score ≥ 10); (iv) fluent in Spanish and able to provide written consent. Exclusion criteria were: (i) severe medical or mental disorder; (ii) cognitive impairment; (iii) psychological treatment in the past year; (iv) inability to attend group sessions; (v) scheduled surgery; (vi) contraindications to MRI.

**FIGURE 1:**
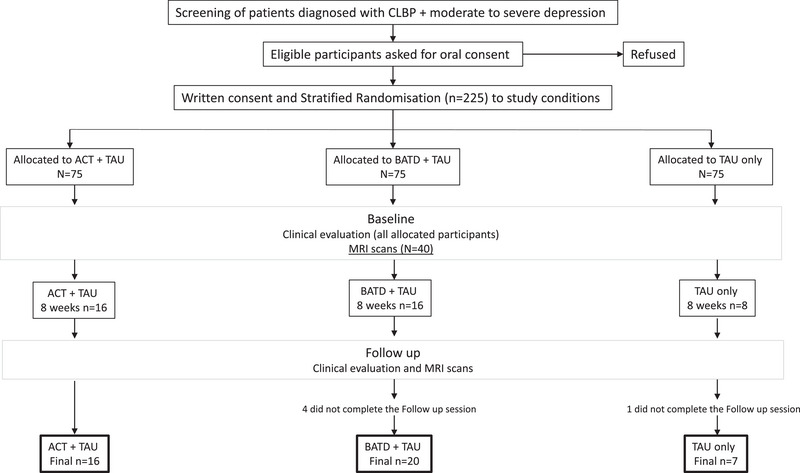
Flowchart of experimental design for the IMPACT neuroimaging sub study. Figure adapted from IMPACT study protocol.

For the sub‐study, only participants who reported a diagnosis of depression according to the Composite International Diagnostic Interview‐depression section (CIDI) (World Health Organization 1997) were invited to participate. In addition, 40 healthy controls (HCs) were recruited for MRI measurements only, excluding those with: age < 18 or > 70, left‐handed, psychiatric disorders, neurological disorders, alcohol/substance dependence, brain damage, or intellectual disability. HCs were recruited by word of mouth from hospital staff or friends/relatives of hospital staff and by poster advertisement at the PSSJD. A flowchart of the imaging sub study design is shown in Figure 1.

### 2.3 Procedure

After eligibility was assessed by health psychologists and written consent was obtained, participants were randomized to the treatment arm, blinded to allocation (see Measures section). Participants who met the eligibility criteria attended a baseline face‐to‐face interview at the hospitals with trained clinical psychologists. Before providing informed consent and administering the battery of self‐report measures, participants were informed of the study's purpose and confidentiality agreements. They were also notified that they were free to withdraw from the study at any time with the assurance that they could continue to receive their usual treatment. Baseline measures were collected, including demographic and clinical information, pain intensity ratings, pain‐related outcomes, and MRI. After 8 weeks of treatment, participants returned for follow‐up assessments of pain ratings and clinical variables.

Randomization of participants to treatment arms was performed after the completion of baseline clinical assessments as recommended by the CONSORT guidelines. Following Ost's recommendations, participants were randomly assigned to ACT and BATD therapists to control possible therapist effects on the outcome. This allocation process was performed by a statistician who was not involved in any other research or treatment delivery procedures. Participants were assigned a list of alphanumeric codes and then randomly assigned to groups using SPSS (v26). In this process, stratified randomization was performed considering baseline pain (NRS; ≥ 7 points out of 10 points) and depressive symptom (PHQ‐9; ≥ 15 points out of 27 points) scores to ensure comparable clinical severity ratings between groups.

### 2.4 Interventions

#### 2.4.1 TAU

It was provided to patients by general practitioners, typically including analgesics, antidepressants, and referral to pain clinics for more specific treatment and monitoring in the more severe cases. For all participants, TAU remained constant throughout the trial.

#### 2.4.2 ACT + TAU

ACT is a new form of CBT (Fenn and Byrne 2013; Hayes et al. 2011). In general terms, this therapy aims to promote action‐oriented, non‐avoiding, and goal‐directed thoughts and attitudes, to improve psychological inflexibility, and to become open, aware, and engaged. It is commonly used to treat a variety of psychological problems, such as depression, anxiety, addictions, or obsessive‐compulsive disorder and it has shown to be effective in treating chronic pain (Hughes et al. 2017).

#### 2.4.3 BATD + TAU

It is also a new form of CBT that focuses on the promotion of patterns of behaviors that break cycles of anhedonia and negative reinforcement, usually stemming from avoidance behaviors and lethargic lifestyle (Soucy Chartier and Provencher 2013; Walsh et al. 2021). It is effective in treating depression (Cuijpers et al. 2007) by equipping people with the necessary skills to identify negative patterns of behaviors and choose activities to counteract them, which they can then arrange in order of difficulty and reassess weekly to track their progress and induce positive reinforcement.

ACT and BATD programs were conducted in three waves: October to December 2020 (first wave), February to April 2021 (second wave), and May to July 2021 (third wave). Participants undergoing add‐on treatments (ACT or BATD) attended a weekly group session for 8 weeks lasting 1.5 h. The sessions were run by at least three different trained therapists to control for possible therapist effects on the outcome. The therapists completed a refresher course before the beginning of the treatments to ensure proper adherence to the treatments.

### 2.5 Measures

The neuroimaging sub study used the following measures to test a priori hypotheses. A full list of measures from the main RCT can be found in the study protocol (Sanabria‐Mazo et al. 2020).

#### 2.5.1 Demographic and Clinical Measures

Age, medication, years since CLBP and depression diagnoses, comorbid conditions, and family history of mental illness were recorded.

#### 2.5.2 Self‐Report Measures

These were collected at baseline and follow‐up to assess treatment outcomes.

- Numerical Rating Scale (NRS) (Chiarotto et al. 2018): Participants rated their average back pain intensity over the past week from 0 (“no pain”) to 100 (“worst pain imaginable”).
- Depression Anxiety Stress Scales‐21 (DASS‐21) (Lovibond and Lovibond 1995): This instrument reliably distinguishes between depression, anxiety, and stress features. We used the depression scale to assess depression symptoms, with scores ranging from 0 (no depression) to 21 (severe depression). The scale demonstrated adequate internal consistency in this sample (*α* = 0.82), and the Spanish validation (Bados et al. 2005) showed satisfactory convergent and discriminant validity.
- Pain Catastrophizing Scale (PCS): (Sullivan et al. 1995) This instrument measures rumination, magnification, and helplessness concerning pain. The PCS is composed of 13 items, which are answered on a 0 (“never”) to 5 (“almost always”) scale. We used both total and subscale scores. The scale demonstrated excellent internal consistency in this sample (*α* = 0.90), and the Spanish validation (García‐Campayo et al. 2008) showed adequate convergent validity.
- Psychological Inflexibility in Pain Sale (PIPS) (Wicksell et al. 2010): This instrument is usually employed to assess the effects of CBT on avoidance, cognitive fusion, value orientation, and acceptance. This measure was added following pre‐registration for the trial. The PIPS is composed of 12 items, which are answered on a 1 (“never true”) to 7 (“always true”) scale. The scale showed excellent internal consistency in this sample (*α* = 0.85), and the Spanish validation (Rodero et al. 2013) showed adequate construct validity.

#### 2.5.3 MRI Data Acquisition and Preprocessing

Imaging was performed on a 1.5 T GE Signa whole‐body MR scanner with an 8‐channel, 8NVHEAD‐A head coil. All participants underwent a T1‐weighted 3D structural scan via a fast spoiled gradient echo (FSPGR) pulse sequence with 70 slices, slice thickness = 2 mm, repetition time = 12 ms, echo time = 3.76 ms, and flip angle = 20°. Participants also underwent a functional rsBOLD scan per session using a single echo, echo planar imaging sequence (repetition time = 2225 ms, echo time = 40 ms, flip angle = 90°, matrix size 64 × 64, 26 transverse slices with thickness = 4 mm with 1 mm gap, voxel size = 3.75 × 3.75 × 5). Initial visual inspection suggested no obvious artifacts. Preprocessing was performed with FMRIB Software Library (FSL) (2012) version 5.0.11. MCFLIRT was used for 3D volume realignment on rsBOLD images and FSL BET was used for skull stripping. The mean image of each 4D scan was calculated and coregistration parameters to the T1 scan were estimated using FLIRT. FNIRT was used for non‐linear warping to normalize the structural images to a template, and the warping parameters were used to normalize the mean functional scans. 4D images were spatially smoothed with an 8 mm Gaussian kernel. Denoising of rsBOLD images was performed using ICA‐AROMA, and white matter (WM) and cerebrospinal fluid (CSF) signals were regressed using a general linear model (GLM). Frame‐to‐frame shifts (i.e., average volume‐to‐volume movement) were calculated using BRAMILIA tools (https://users.aalto.fi/~eglerean/bramila.html), and scan‐nulling regressors for volumes with shifts > 0.5 mm, which was considered the threshold for excessive motion, were added to the GLM. The purpose of this step was to systematically account for motion‐related confound while preserving the maximum amount of data. The 4D scans were high‐pass filtered (200 s) and normalized to Montreal Neurological Institute (MNI) space using affine and non‐linear warping parameters with trilinear interpolation.

### 2.6 Data Analysis

#### 2.6.1 Clinical Data

We performed a one‐way analysis of variance (ANOVA) on demographic and clinical measures (age and years from CLBP diagnosis) and self‐reported measures (DASS‐21‐D, NRS, PCS, and PIPS) at baseline to ensure compatibility across the three treatment arms. We also explored within‐group changes following treatment using paired samples *t*‐tests for all self‐report measures of interest with SPSS v.26 (https://www.ibm.com/uk‐en/products/spss‐statistics).

### 2.7 Neuroimaging Data

#### 2.7.1 Regions of Interest

To examine the FC of the SN, we created a 6‐mm sphere on both anterior insulae, using coordinates from a recent meta‐analysis that identified this region as a robust biomarker for chronic pain (Ferraro et al. 2022). In this case, we opted for spherical seeds to focus on the core region shown to be involved in clinical pain in a region of considerable size and high functional heterogeneity. For the SMN, we followed the approach of Mao et al. (2022) and selected anatomical regions of interest (ROIs) for the precentral and postcentral gyri from the Harvard‐Oxford cortical/subcortical probabilistic structural atlases (Frazier et al. 2005), which provide a population‐derived account of brain regions. To ensure specificity, probabilistic ROIs were thresholded to include voxels with the top 50% values and binarized. For FC of the mesolimbic pathway, it was critical to choose a seed that accounted for the ventral striatum as a separate entity, as a main relay of the mesolimbic system. Consequently, we used an anatomical ventral striatum seed from Fujiwara et al. (2012).

#### 2.7.2 First‐Level Analyses

**FIGURE 2:**
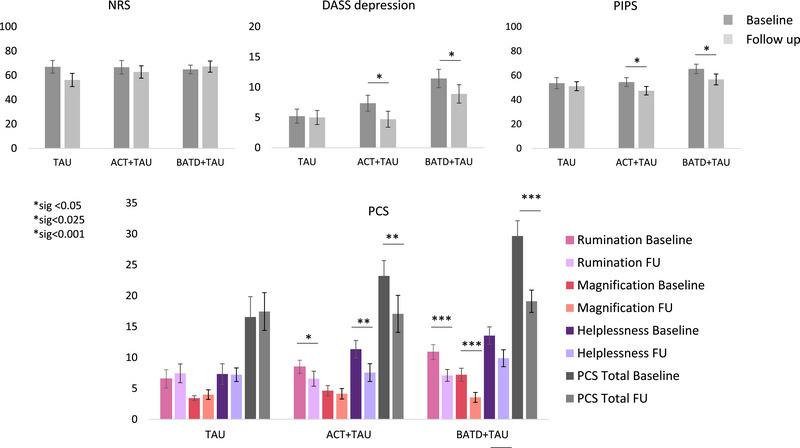
Summary of results from psychometric data. Top panel depicts group results at baseline and follow up, separately for reported pain (NRS measures), reported depression measures (DASS depression) and psychological inflexibility for pain (PIPS). Paired‐samples *t*‐test across all measures within each treatment arm revealed significant reductions in depression and PIPS measures following ACT + TAU and BATD + TAU, and no significant changes in reported pain. Bottom panel depicts results for total pain catastrophizing scores (PCS, grey) as well as for each one of the PCS subscales, namely, rumination, magnification, and helplessness (pink, salmon, and purple shades, respectively). Paired‐samples *t*‐test revealed significant overall reductions in pain catastrophizing following ACT + TAU and BATD + TAU, which was also reflected when observing rumination and helplessness alone in the ACT + TAU arm and when observing rumination and magnification alone in the BATD + TAU arm.

For each participant and network, we calculated voxel‐wise Pearson's *r* correlations (Penny et al. 2011) between the mean time series across seeds and the remaining voxels in the brain within the grey matter (GM), using an explicit GM mask. Correlation maps were Fisher *Z*‐transformed for group analyses. FC maps were calculated in MATLAB version 9.5.0 (R2010a). Before performing second‐level analyses, we confirmed the correct positioning of seeds and the accurate distribution of FC within each network using a one‐sample *t*‐test across participant and healthy control groups separately and for each network in SPM12. The FC network distributions and seed positions are shown in Figure 2.

#### 2.7.3 Second‐Level Analyses

rsBOLD images were analyzed within a mass univariate GLM approach in SPM12, with an initial clustering threshold set at *p* < 0.001. For ROI analyses, small volume corrections (SVCs) were applied to each contrast within the network of interest. Independent SN, SNM, and ML masks for SVC were obtained from one‐sample *t*‐test results for each network from HC, thresholded to include voxels with *z* values greater than 5 to ensure conservative SVC areas. Family‐Wise Error (FWE) correction at peak extent *p* < 0.05 was applied to post‐SVC results, and FEW‐corrected cluster extent values were used for post hoc exploratory whole‐brain analyses. We first explored whole‐brain FC differences between networks and the rest of the brain in participants compared to HC using an independent sample *t*‐test. To explore relationships between FC and CLBP + depression symptoms, we created multiple regression models for each FC map separately across all participants, using NRS, DASS‐21‐D, PCS, and PIPS as regressors. The NRS and DASS‐21‐D regressors were analyzed together in one model to explore the relationships between FC and pain independent of depression scores.

We also assessed whether FC could predict treatment outcomes within each arm and network, calculating delta scores (baseline—follow‐up) for the NRS and other measures as regressors. Finally, we compared FC and delta pain scores between ACT and BATD by estimating separate regression models for each FC network, using delta NRS as a regressor, and testing whether the linear relationship between FC and delta pain scores differed across groups by comparing their slopes. All models included age as a nuisance covariate.

#### 2.7.4 Calculation of Effect Sizes

Since our final samples were smaller than initially planned, we calculated a posteriori approximations of Hedge's *G* (Hedge's *G*_a_) voxelwise maps from the *t* maps of all main contrasts of interest (Bossier et al. 2019). Unlike Cohen's *d*, Hedge's *G*_a_ helps to control for common overestimations of effect sizes in samples below 20 (Hedges 1981).

## 3 Results

### 3.1 Baseline Demographic and Clinical Measures

Forty participants completed the baseline assessments (sample sizes: TAU = 9, ACT+TAU = 15, BATD + TAU = 16). Thirty‐two of them completed all visits (TAU = 9, ACT+TAU = 14, BATD + TAU = 9). HC data (*n* = 14) were included to ensure age‐matching. Clinical and demographic characteristics are summarized in Table 1. One‐way ANOVAs showed no significant baseline differences across groups for age, DASS‐21‐D, NRS, and PIPS. PCS total and magnification subscale scores were significantly higher in the TAU‐only group compared to the BATD group, but not across treatment groups. After 8 weeks of intervention, both the ACT and BATD groups showed significant reductions in DASS‐21‐D, PIPS, and PCS (Figure 2). There was no significant group reduction in NRS scores, although considerable variability was observed (Figure S1). The TAU‐only group showed no significant changes.

**TABLE 1:** Summary of demographic, clinical and self‐report measures included in the neuroimaging sub study.

	ACT + TAU (*N* = 14)		BATD + TAU (*N* = 9)		TAU (*N* = 9)	
	Baseline Mean (SD)	Follow‐up Mean (SD)	Baseline Mean (SD)	Follow‐up Mean (SD)	Baseline Mean (SD)	Follow‐up Mean (SD)
Age	58.63 (6.24)	—	56.97 (4.77)	—	54.37 (8.33)	—
Years from CLBP diagnosis	11.28 (10.44)	—	11 (9.11)	—	9.33 (5.24)	—
Pain NRS (0–100)	66.64 (20.67)	62.78 (18.88)	64.88 (10.69)	67.22(13.69)	67 (15.62)	56.22 (16.3)
DASS depression	7.35 (4.39)	4.71 (5.41)	11.44 (4.55)	8.88 (5.46)	5.22 (3.49)	5 (3.9)
PIPS	54.5 (13.59)	47.35 (13.03)	65.63 (11.68)	56.66 (13.36)	53.55 (13.70)	51.11 (11.02)
PCS Total	23.21 (9.25)	17.07 (11.22)	29.66 (7.39)	19.11 (5.41)	16.55 (9.9)	17.44 (9.16)
PCS Rumination	8.5 (4.01)	6.57 (4.53)	10.88 (3.62)	7.11 (2.89)	6.55 (4.44)	7.44 (4.55)
PCS Magnification	4.64 (3.1)	4.14 (3.15)	7.22 (3.15)	3.55 (2.40)	3.44 (1.13)	4 (2.34)
PCS Helplessness	11.35 (5.28)	7.57 (5.38)	13.55 (4.24)	9.88 (4.10)	7.33 (4.97)	7.22 (3.3)

Abbreviations: ACT = acceptance and commitment therapy; BATD = behavioral activation for the treatment of depression; CLBP = chronic low back pain; DASS = Depression Anxiety Stress Scale; NRS = numerical rating scale; PCS = Pain Catastrophizing Scale; PIPS = Psychological Inflexibility for Pain Scale; TAU = treatment as usual.

### 3.2 FC Differences Between CLBP and HC Groups

**FIGURE 3:**
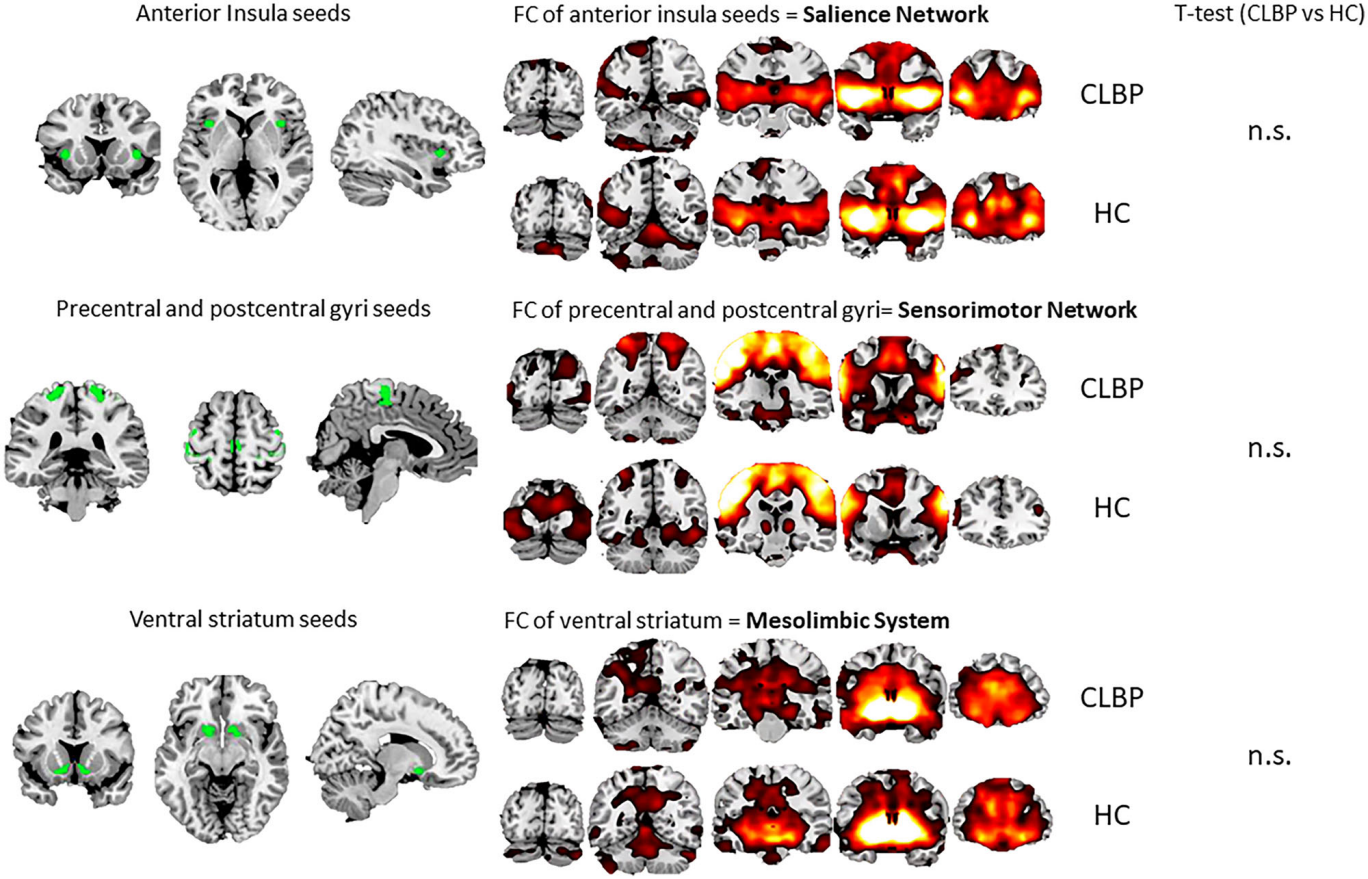
One *t*‐test results for each FC network of interest across CLBP patients and HC. Left panel depicts, from top to bottom, seeds chosen to extract BOLD signals from insula bilaterally, precentral and postcentral gyri bilaterally, and ventral striatum bilaterally. Central column depicts significant voxels following one‐sample *t*‐test across individual FC maps, arising following the calculation timeseries correlations between seeds and the rest of the voxels covering the brain. Results indicated that at baseline, anterior insulae were functionally connected with areas considered part of the salience network, precentral and postcentral gyri were functionally connected to main nodes of the sensorimotor network, and ventral striatum was functionally connected to areas across the mesolimbic system. Independent‐samples *t*‐test revealed that there were no significant differences between CLBP patients and HC across all FC networks of interest. All group results are significant following FWE correction for multiple comparisons. n.s. = non‐significant.

One sample *t*‐tests on baseline FC maps for the SN, SMN, and ML pathways showed the expected FC distribution across the brain in both CLBP and HC groups (Figure 3). Independent samples *t*‐tests revealed no significant differences in FC networks between CLBP participants and HC.

### 3.3 Baseline Relationship Between FC and CLBP Symptoms

**FIGURE 4:**
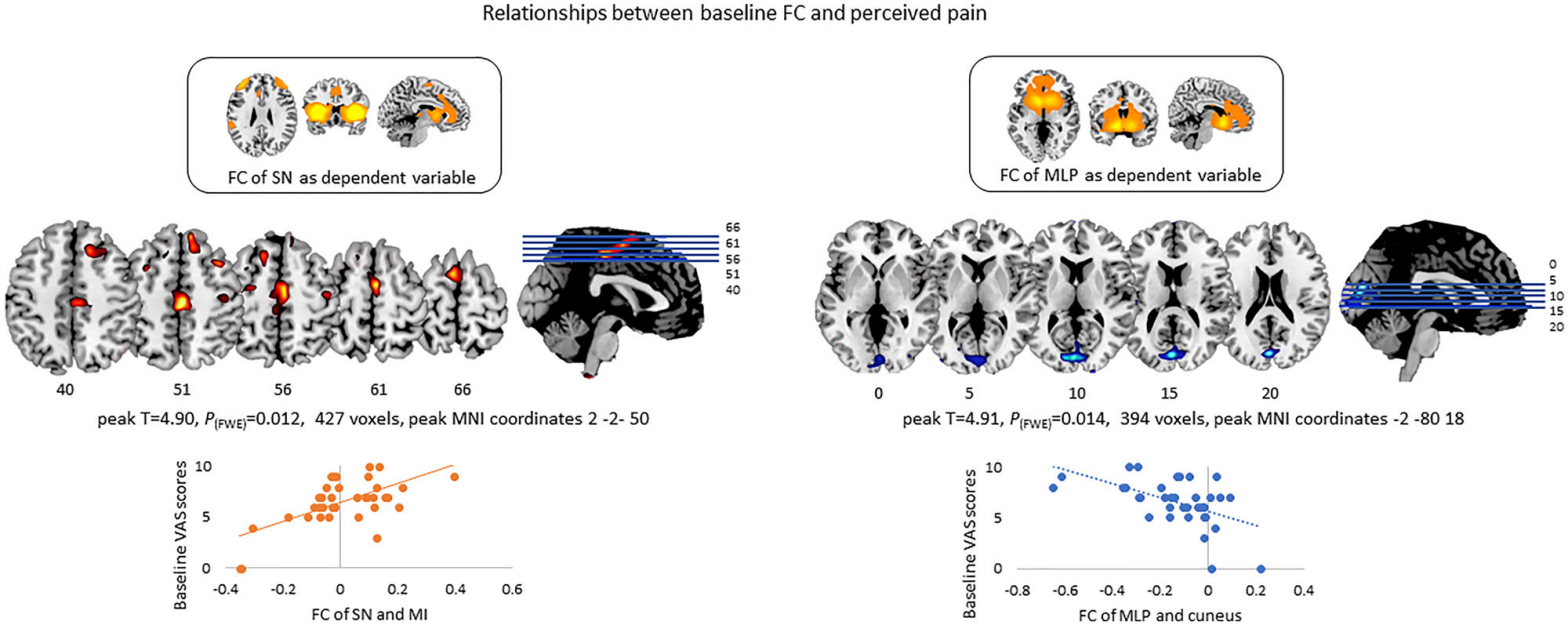
Results from multiple regression models between FC of each network of interest (dependent variable) and clinical measures (regressors). When taking the FC of the SN as dependent variable (left panel) a positive correlation between baseline VAS scores and the connectivity of the network with the primary motor cortex was observed (e.g., the higher the reported pain at baseline, the stronger the connectivity). When taking the FC of the MLP as dependent variable (right panel) a negative correlation between baseline VAS scores and FC of the network and the cuneus was observed (e.g., the more reported pain at baseline, the weaker the connectivity).

Exploratory analysis revealed that baseline NRS scores correlated positively with SN FC to primary motor cortex and negatively with MLP FC to cuneus (Figure 4). In other words, the higher perceived pain intensity at baseline was associated with stronger SN‐SMN FC and weaker MLP‐cuneus FC. No relationship was found between SMN FC and any of the clinical measures. In addition, baseline DASS‐21‐D, PIPS, and PCS scores did not show significant correlations with FC in any of the networks of interest.

### 3.4 FC as Predictor for Treatment Outcome

**FIGURE 5:**
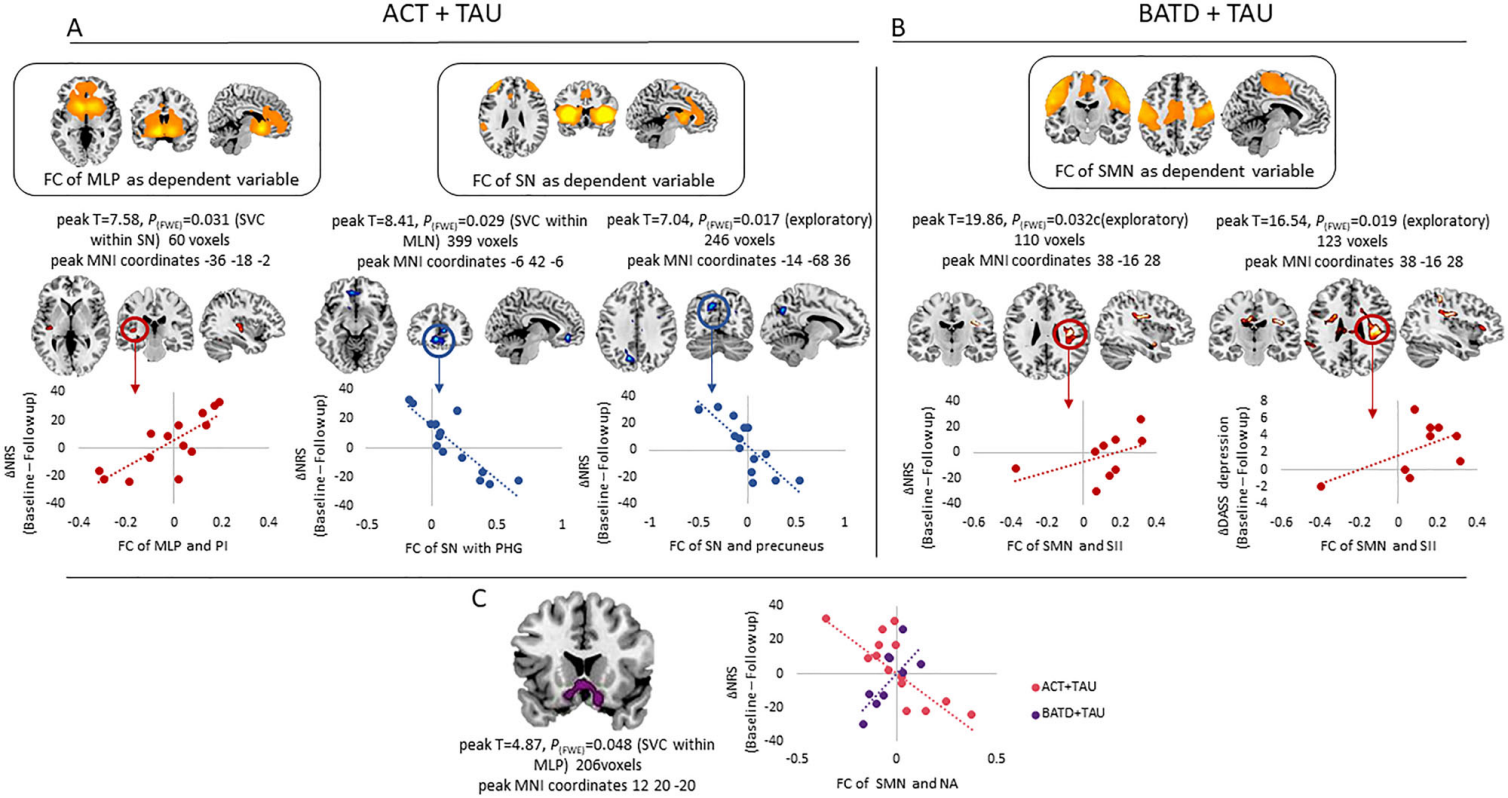
Significant results from multiple regression models between FC of each network of interest (dependent variable) and treatment outcome according to delta scores on clinical measures (regressors). Top panel depicts results separately for ACT + TAU (panel A, left) and BATD + TAU (panel B, right). For each significant result, *X*‐axes represent FC values between dependent variable and significant clusters and *Y*‐axes represent changes in self‐reported measures following treatment. For self‐reported measures, positive values reflect reductions following treatment, and vice versa. Panel C depicts the differences in correlations between changes in self‐reported pain and FC of the SMN and NA across active treatment arms, showing contrasting relationships (e.g., larger analgesic effects correlated with weaker baseline FC for the ACT + TAU group, but with stronger baseline FC in the BATD + FC group).

We examined whether baseline FC of our networks of interest predicted responses to ACT and BATD for pain and depression symptoms. Change in NRS score (i.e., delta NRS: baseline—follow‐up NRS scores) was considered an outcome measure for the treatment of pain. Within the ACT group (Figure 5A), baseline FC of the ML pathway and the posterior insula (PI) correlated positively with delta NRS, indicating that stronger connectivity predicted greater improvement with ACT. Conversely, FC of the ML pathway and paracingulate gyrus correlated negatively with delta NRS, with stronger baseline connectivity indicating less benefit. Finally, whole‐brain analyses showed that FC of the SN and precuneus also correlated negatively with delta NRS, with stronger connectivity predicting smaller improvements. No significant relationships were found between FC and changes in depression scores for ACT. For the BATD group (Figure 5B), FC of the SMN and postcentral gyrus extending to the secondary somatosensory cortex (SII) correlated positively with delta NRS and delta DASS depression scores, with stronger connectivity predicting greater improvements in pain intensity and depression. No other significant relationships between FC and clinical outcomes were observed in this group.

When comparing the regression slopes between FC and delta NRS (Figure 5C), we found a significant difference in the relationship between FC of the SMN and bilateral nucleus accumbens (NA) across groups. For ACT, greater FC predicted less improvement (negative slope), whereas for BATD, greater FC predicted more improvement (positive slope). No significant differences in regression slopes were observed for delta NRS in the other two networks of interest.

**FIGURE 6:**
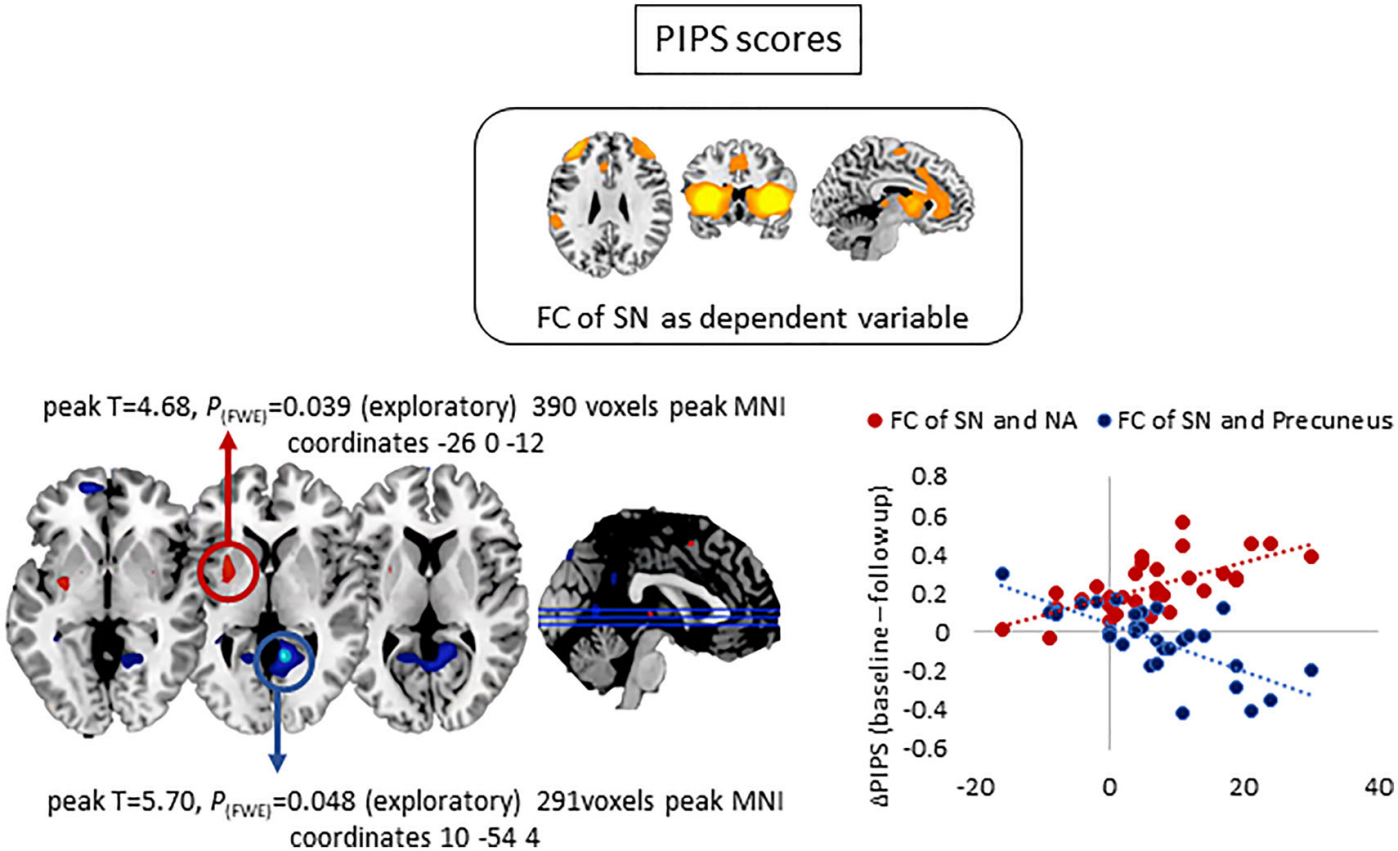
Significant results from multiple regression models between FC of SN (dependent variable) and delta PIPS scores across ACT + TAU and BATD + TAU treatment arms. Results indicated that larger improvements in psychological inflexibility following treatment corresponded to stronger baseline FC of the SN and the NA (red) as well as weaker baseline FC between the SN and precuneus (blue).

We also explored whether baseline FC of the SN was related to changes in PIPS and PCS scores. As the primary aim of ACT and BATD (and CBTs in general) is to improve psychological inflexibility and reduce catastrophizing thoughts, we first analyzed this in all CLBP participants. Regression analyses showed a positive relationship between SN and left putamen FC and delta PIPS (stronger FC at baseline associated with more flexibility at follow‐up) and a negative relationship between SN connectivity with the posterior cingulate cortex (PCC) and delta PIPS (stronger FC at baseline associated with less flexibility at follow‐up) (Figure 6). These relationships did not hold when analyzed by group. Finally, delta PCS scores did not correlate with either SN or SMN FC across the sample.

## 4 Discussion

This study investigated the relationship between FC in the SN, SMN, and MLP with pain intensity, depression, and pain‐related symptoms in individuals with CLBP and comorbid depression. It also explored how symptom changes after ACT and BATD correlated with baseline FC within these networks. As hypothesized, stronger baseline FC in the salience and sensorimotor networks was associated with higher pain intensity, while connectivity in the MLP and precuneus was linked to lower pain. Both ACT and BATD led to reductions in depression, psychological inflexibility, and pain catastrophizing, with specific patterns of brain connectivity predicting treatment outcomes. Together, our data provide new insights into the overlap between neural correlates of these psychological processes and the pathophysiology of CLBP. We will now discuss which brain connectivity patterns may be responsible for improvement after ACT and BATD.

At baseline, we found a positive correlation between FC of the SN and primary motor cortex (part of the SMN) with pain levels, supporting previous research linking sensorimotor circuits to pain processing (Kim et al. 2019; Isenburg et al. 2021). This association may also relate to depression, as sensorimotor modulation plays a role in higher‐order pain processing, emotion regulation, and prosocial behavior (Riečanský and Lamm 2019; Canbeyli 2010). In line with this, stronger connectivity between the SII and the rest of the SMN predicted greater reductions in depression following BATD. We also observed a negative relationship between baseline pain and FC between the MLP and the cuneus. Altered connectivity in these regions, particularly between the cuneus and mesolimbic areas, has been related to suicidal thoughts (Wei et al. 2018) and childhood trauma (Luo et al. 2022) in depression. Similarly, FC of the cuneus and prefrontal and salience areas is greater in relapsing alcohol abusers than in abstainers (Wang et al. 2018), suggesting that maladaptive patterns may contribute to pain intensity in CLBP. In the ACT group, greater pain intensity reduction was associated with stronger baseline FC between the MLP and the posterior insula, indicating a potential role of sensorimotor processing in modulating pain sensitivity (Uddin et al. 2017; Segerdahl et al. 2015; Bergeron et al. 2021). In contrast, FC between the SN and both the parahippocampal gyrus and the precuneus, both part of the default mode network (DMN) (Schulz et al. 2010; Utevsky et al. 2014) related to poorer pain relief following ACT. This suggests that efficient switching between task‐relevant (SN) and irrelevant (DMN) states requires optimal communication between both networks and insufficient SN‐DMN connectivity may hinder attentional shifts away from painful stimuli.

As hypothesized, FC of the SMN was related to responses to BATD, with stronger FC between SII and the rest of the SMN corresponding to greater pain reduction following BATD. Previous research has shown maladaptive SI and SII reorganization during mechanical stimulation in CLBP (Hotz‐Boendermaker et al. 2016). Induced sensorimotor neuroplasticity has been proposed as a mechanism for pain relief through cognitive therapies in CLBP by eliminating danger cues and promoting motor imagery and specific exercises (Brumagne et al. 2019). The SII, which integrates somatosensory input and emotional responses from the insula (Orenius et al. 2017), may serve as a valuable target in this process. Our findings provide preliminary evidence supporting the role of BATD in promoting physical activation. We speculate that baseline sensorimotor integrity may indicate the potential for BATD to induce neuroplasticity, potentially reversing maladaptive somatosensation.

Comparing ACT and BATD revealed contrasting mechanisms based on baseline FC. Here, a strong FC between SMN and NAc is related to larger pain relief for BATD, but poorer outcomes for ACT. The NAc, a key hub in the dopaminergic system, is thought to mediate motivational, affective, and self‐regulatory aspects of chronic pain (Benarroch 2016) and sensorimotor function. BATD may induce sensorimotor plasticity via the NAc, with changes in afferent signaling to the NAc evoking analgesia through dopaminergic disinhibition via the ventral tegmental area (Yang et al. 2018; McCutcheon et al. 2019). Thus, a greater baseline FC may predict better BATD outcomes. In contrast, ACT focuses on motivational and cognitive changes via other limbic areas, such as the nigrostriatal pathway (Han et al. 2018; Patel et al. 2014), where maladaptive SMN‐NAc connectivity could hinder pain relief.

Behaviorally, third‐wave CBTs as add‐ons to TAU significantly reduced depression, pain catastrophizing, and psychological inflexibility about pain intensity at 8 weeks compared with TAU alone. This supports the efficacy of multicomponent treatment plans for the management of CLBP and other chronic pain conditions (Hylands‐White et al. 2017) and emphasizes the importance of early referral to multidisciplinary pain clinics. However, no reduction in NRS pain intensity scores was observed in either group, consistent with the fact that CBTs are not designed to treat pain intensity directly. The variability in pain intensity scores after 8 weeks was expected and crucial to understanding their relationship with FC patterns. Therefore, the lack of group pain intensity reduction does not affect the interpretability of our findings.

Improvements in cognitive flexibility were associated with the strong baseline FC of the SN and NAc. Maladaptive salience‐striatal connectivity concerning cognitive flexibility has been described in obsessive‐compulsive disorder (Tomiyama et al. 2019) and major depressive disorder (Peters et al. 2016). Our data suggest that this pathway may also mediate cognitive flexibility for pain in CLBP. Deficient synaptic plasticity between the SN and NAc may preclude flexible updating of reward‐based behaviors in chronic pain (Chang and Creed 2022) and therefore more effective baseline communication between both regions may facilitate better outcomes measured via PIPS. The precuneus, which plays a key role in consciousness and attention shifting (Cavanna and Trimble 2006), showed decreased FC with the cognitive control network, which has also been related to depression (Hwang et al. 2015). Based on this, we speculate that strong synchrony between the precuneus and SN might facilitate engagement with negative salient interoceptive stimuli while hindering attention shifts towards other thoughts/stimuli/behaviors, making cognitive flexibility improvements harder to achieve.

Finally, we found no correlation between baseline or delta DASS depression scores, PCS scores, and FC across participants. Although unexpected, this may reflect that brain correlates of depressive and emotionally negative symptoms in CLBP are better understood in terms of their “quality” (e.g., primary symptoms) rather than their “intensity” (e.g., severity), a hypothesis that requires further exploration. We also found no significant differences within our networks of interest between CLBP participants and HC, which, together with our other results, suggest that the underlying pathophysiological mechanisms of CLBP may reside in erratic internetwork communication and feedback, rather than within network dysfunctions, especially considering heterogeneous attributes of CLBP and depression phenotypes.

This study had several limitations. First, smaller than planned sample sizes due to participant withdrawals may have reduced the power and sensitivity to identify significant findings. Nonetheless, we calculated effect size Hedge's *G* estimations, which control for effect size inflation in small samples. Most effect sizes were large (Figure S2), providing some confidence in the magnitude and power of the results. However, replication in an independent sample is recommended. Second, potential shifts in TAU during the trial may have added variability to the responses. Although this is common in clinical research due to ethical obligations to allow medication use, it could be addressed in future studies by stratifying participants based on concomitant medication. Larger sample sizes and closer monitoring of TAU variability would be required, and mobile‐based electronic data capture may provide a feasible solution for adherence tracking (Sarabi et al. 2016).

### 4.1 Clinical Implications

The findings of this study have important clinical implications, particularly in the realm of personalized medicine. Identifying specific baseline FC patterns that predict treatment response could help tailor interventions to individual participants, potentially improving treatment outcomes. For instance, participants with strong baseline FC between the SMN and NAc may benefit more from BATD, while those with different connectivity patterns might respond better to ACT. This suggests that FC biomarkers could be used to guide treatment personalization, allowing clinicians to select the most appropriate therapy based on a participant's unique brain connectivity profile. Additionally, integrating mobile‐based electronic data capture for monitoring TAU variability and participant adherence could further enhance the precision of treatment plans, providing real‐time insights into treatment progress and facilitating early identification of participants who may need additional support or intervention adjustments.

As previously highlighted, future research should aim to validate these predictive markers in larger and independent samples to enhance the generalizability of the findings. In addition, combining BATD with neuroplasticity‐promoting treatments, such as amphetamine (Tegenthoff et al. 2004) treatment or transcranial magnetic stimulation (Ziemann et al. 2006), could potentially enhance the therapeutic outcomes for CLBP. Future research could also benefit from incorporating longitudinal follow‐ups to assess the long‐term effects of these interventions. These future directions underscore the importance of personalized treatment strategies and the potential for innovative therapy combinations to address the complex interplay between pain and depression in CLBP.

## 5 Conclusion

Our data provides new information regarding the neural correlates of clinical pain and the effects of ACT and BATD for CLBP and depression, suggesting differential mechanisms of action for each therapy. This study highlights the potential of resting‐state fMRI as a predictive marker of treatment outcomes. Our findings contribute to advancing neuroimaging biomarkers into real‐world clinical applications.

## Author Contributions

**Sonia Medina**: data curation, software, formal analysis, methodology, visualization, writing–original draft. **Carlos G. Forero**: conceptualization, funding acquisition, investigation, project administration, methodology, supervision, writing–review and editing. **Juan P. Sanabria‐Mazo**: investigation, writing–review and editing. **Carla Rodríguez‐Freire**: writing–review and editing. **Jaime Navarrete**: writing–review and editing. **Owen G. O'Daly**: methodology, supervision, writing–review and editing. **Matthew A. Howard**: methodology, supervision, writing–review and editing. **Juan V. Luciano**: conceptualization, funding acquisition, investigation, project administration, methodology, supervision, writing–review and editing.

## Ethics Statement

This research was carried out according to the 1964 Helsinki Declaration and was approved by the Ethics Committee of the Fundació Sant Joan de Déu (PIC‐178‐19) and the Hospital del Mar (2019/8866/I).

## Consent

Written informed consent was obtained from all participants. None of the participants received any financial incentive for participating in this study.

## Conflicts of Interest

The authors declare no conflicts of interest.

## Permission to Reproduce Material from Other Sources

No material from other sources was reproduced in this manuscript.

## Reporting Guidelines Compliance

This RCT adhered to the guidelines of the Consolidated Standards of Reporting Trials (CONSORT).

### Peer Review

The peer review history for this article is available at https://publons.com/publon/10.1002/brb3.70380.

## Supporting information



Supporting Information

## Data Availability

Derived data supporting the findings of this study are available from the corresponding author on request.
