# Supplementary material for: Baseline Functional Connectivity of the Mesolimbic, Salience, and Sensorimotor Systems Predicts Responses to Psychological Therapies for Chronic Low Back Pain With Comorbid Depression: A Functional MRI Study
Source: Brain Behav. 2025 Feb 28;15(3):e70380. doi: 10.1002/brb3.70380 (PMC11870833; doi:10.1002/brb3.70380)

**Supplementary Information**

Supplementary figure 1. Individual changes in pain scores across treatment groups following treatment. Results indicate that while paired-samples t-test revealed no significant changes at group level within each treatment group, there was a notable within-group heterogeneity in the responses.


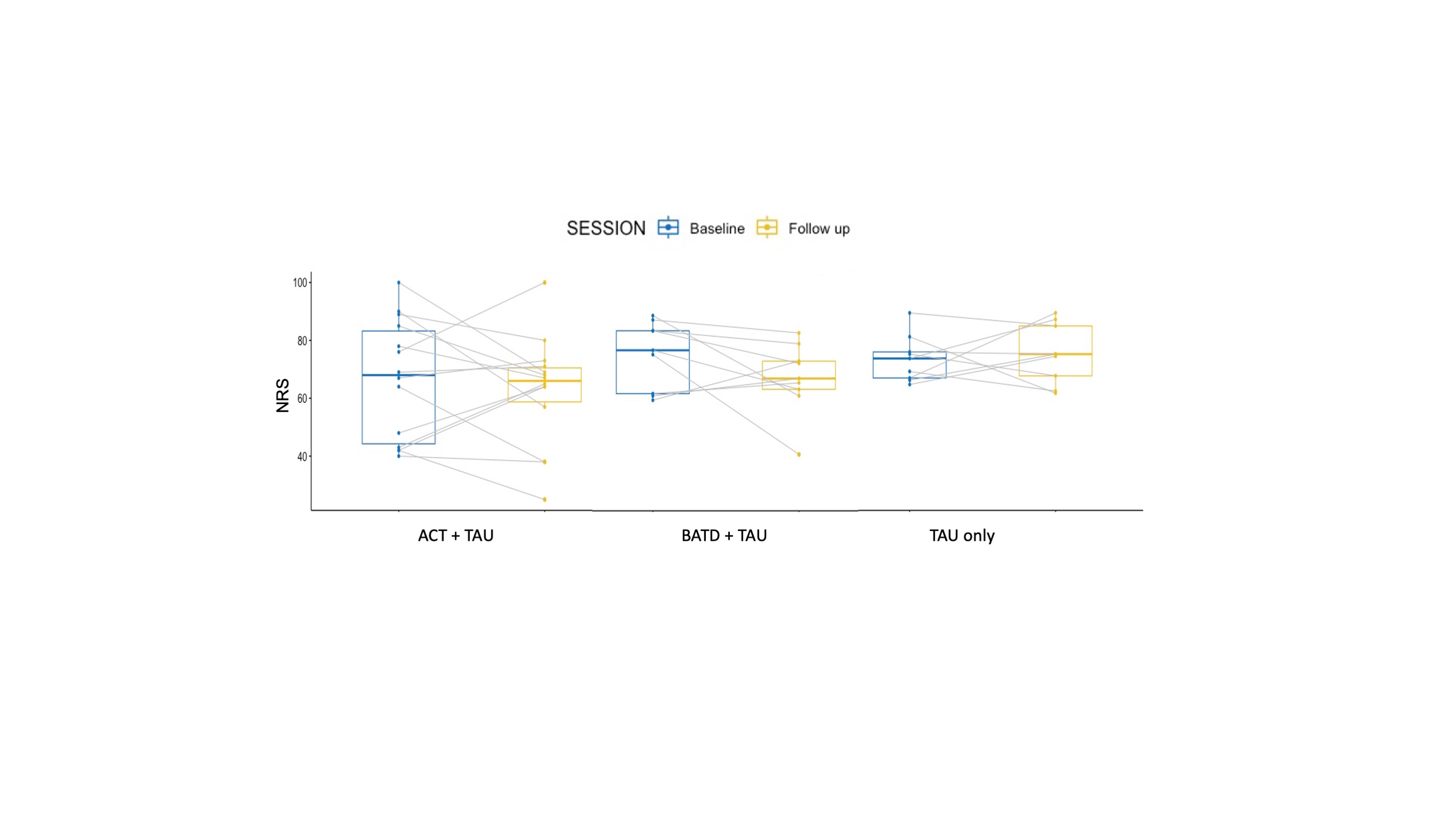


Supplementary Figure 2. Effect size voxelwise maps for each multiple regression model result. All contrasts showed in the result section are depicted above their corresponding Hegde’s G maps (grey background). All results yielded medium (red) to very large (green) effect sizes.


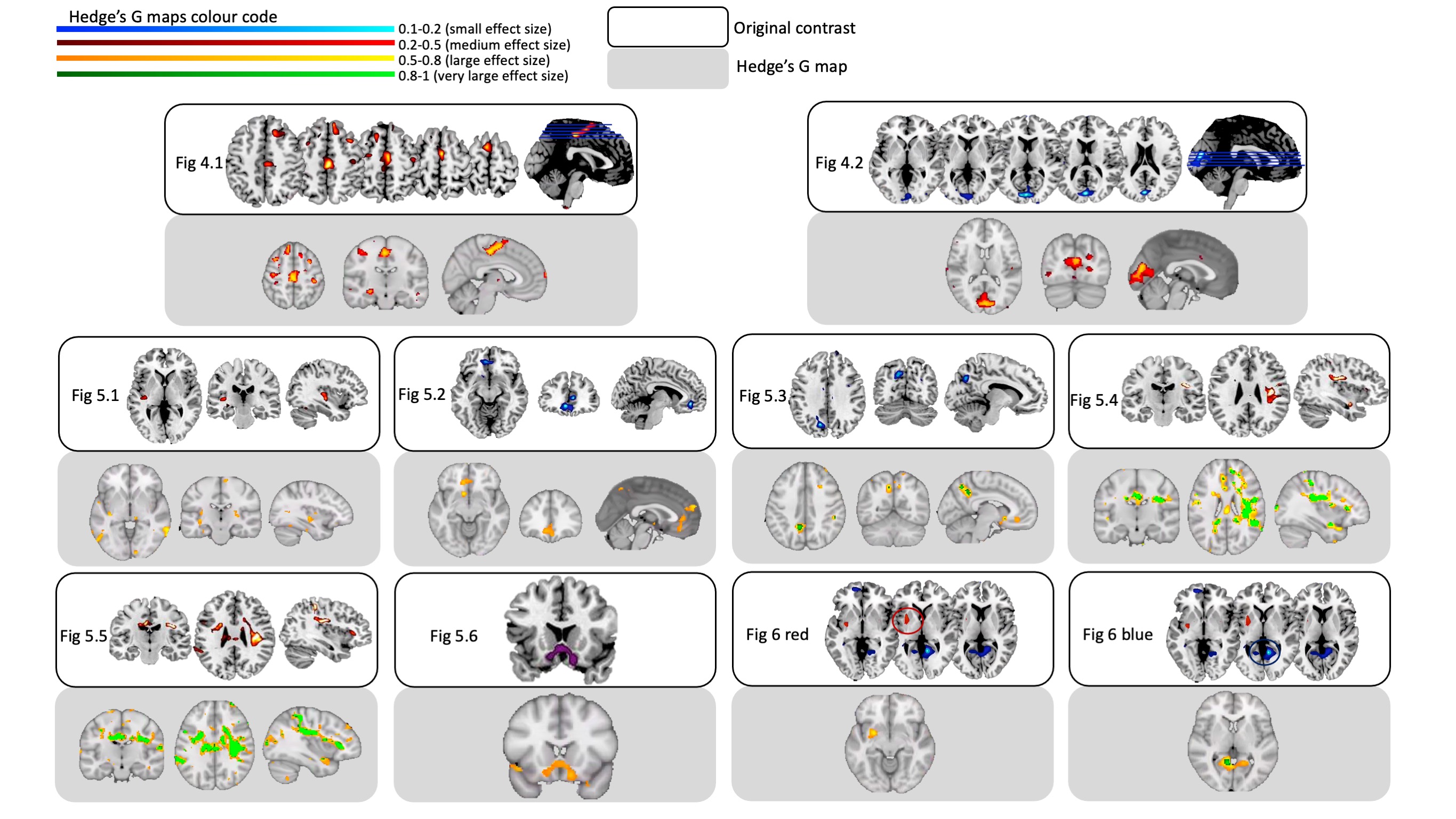

Supplement: Supplementary file 1 — Supporting Information [file BRB3-15-e70380-s001.docx]
